# Supplementary material for: The Effects of a 24-Week Combined Circuit Training and Mobility Program on the Physical Fitness and Body Composition of an Adult Academic Community
Source: Sports (Basel). 2025 Mar 6;13(3):79. doi: 10.3390/sports13030079 (PMC11945721; doi:10.3390/sports13030079)
Supplement: Supplementary file 1 [file sports-13-00079-s001.zip › sports-3368019-supplementary.pdf]

Table S1: Intervention scheme across the 24 weeks.

PF components[1]	Weeks 1-2	Weeks 3-4	Weeks 5-6	Weeks 7-8	Weeks 9-10	Weeks 11-12	Weeks 13-14	Weeks 15-16	Weeks 17-18	Weeks 19-20	Weeks 21-22	Weeks 23-24
CRF	3	2	2	3	2	2	3	2	2	3	2	2
MS	2	3	2	2	3	2	2	3	2	2	3	2
ME	2	2	3	2	2	3	2	2	3	2	2	3
FLEX/BAL	1	1	1	1	1	1	1	1	1	1	1	1
Total station N°	8	8	8	8	8	8	8	8	8	8	8	8

PF: Physical Fitness

CRF: Cardio-Respiratory Fitness

MS: Muscular Strength

ME: Muscular Endurance

FLEX/BAL: Flexibility and Balance

[1] Numbers indicate the exercise stations for each Physical Fitness component.

Figure S1: Circuit Training session example.

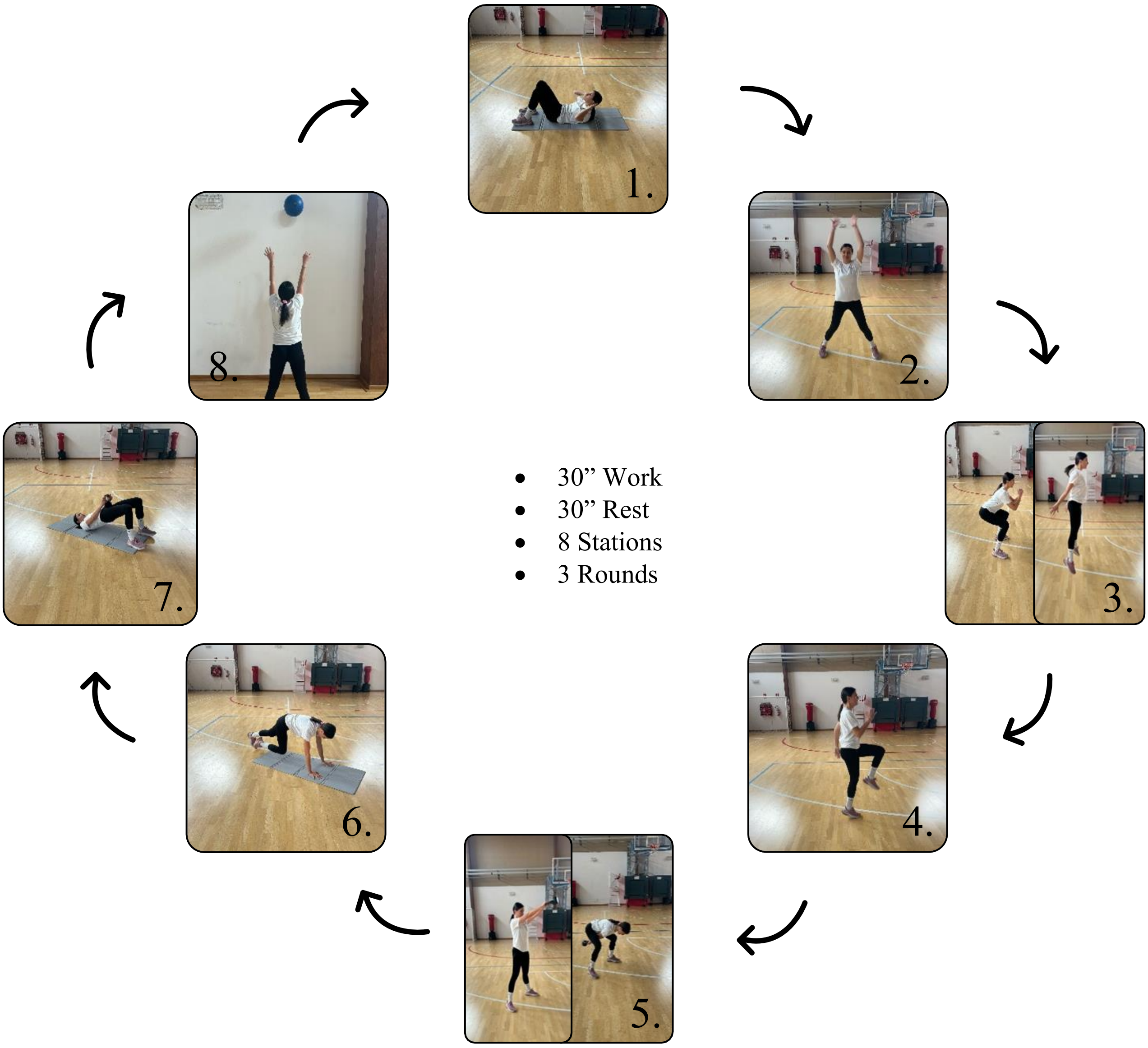

Table S2: Examples of the different stations of CT.

Stations	#1 Example[1]	#2 Example	#3 Example
1	Classic Crunch	Overhead Squat	Burpees
2	Jumping Jack	Step touch	Romanian deadlift
3	Squat Jump	Dead Bug	Triceps kickback
4	High Knee Skip	Side lunges	Shuttle run
5	Swing	Shoulder touch	Walking lunges
6	Mountain Climber	Step touch calf raise	Biceps curl
7	Hip Thrust	Russian Twist	Split jump
8	Wall-ball	Shoulder press	French press

[1] #1 examples are referred to Figure S1.

Figure S2: Mobility session examples (15”–30” or 12–20 repetitions per exercise, with 2–4 sets)

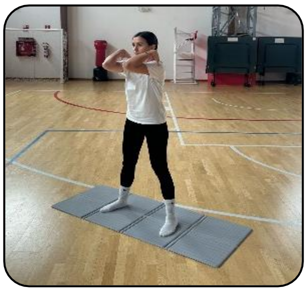
1. Shoulder circles

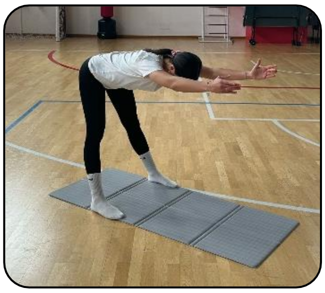
2. Standing forward bend

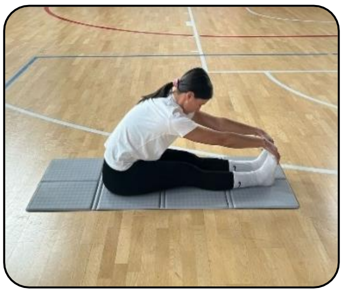
3. Seated forward bend

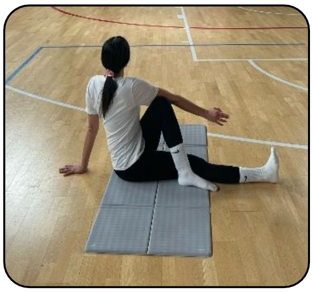
4. Seated Twist Stretch

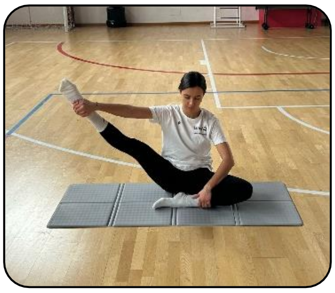
5. Seated leg stretch

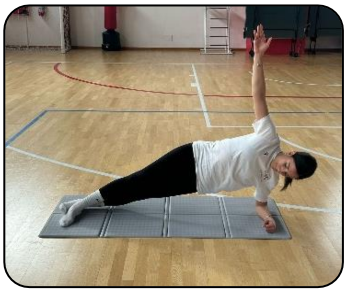
6. Side Plank

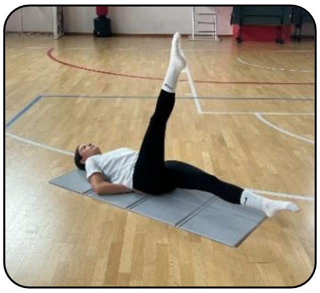
7. Leg scissors

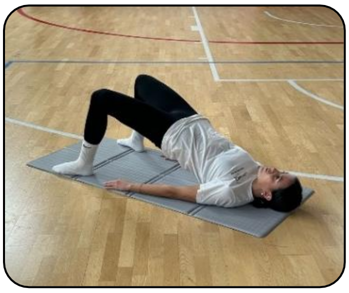
8. Glute bridges

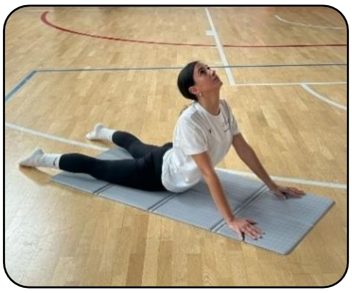
9. Upward facing dog

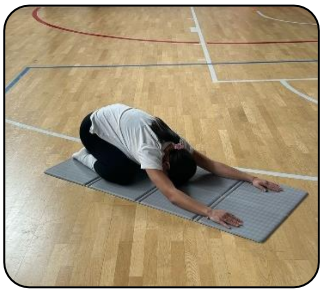
10. Child pose

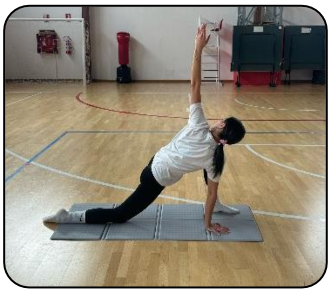
11. Posterior chain stretch

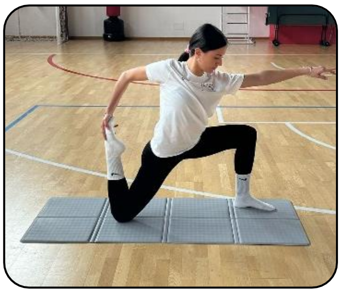
12. Forward lunge stretch

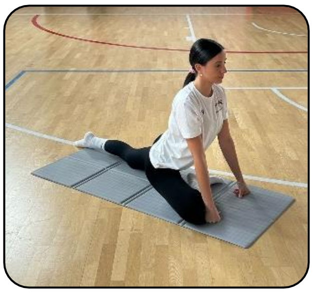
13. Half pigeon

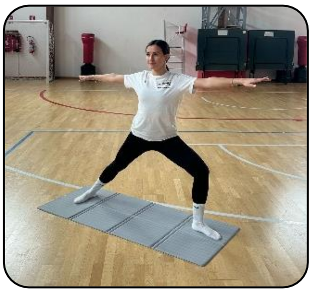
14. Warrior I

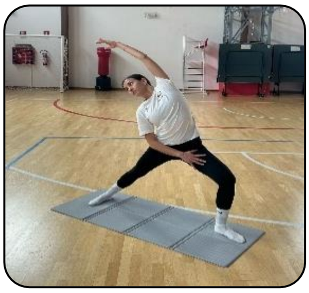
15. Warrior II

Figure S3: Group mean variations across the three timepoints for each significant variable.

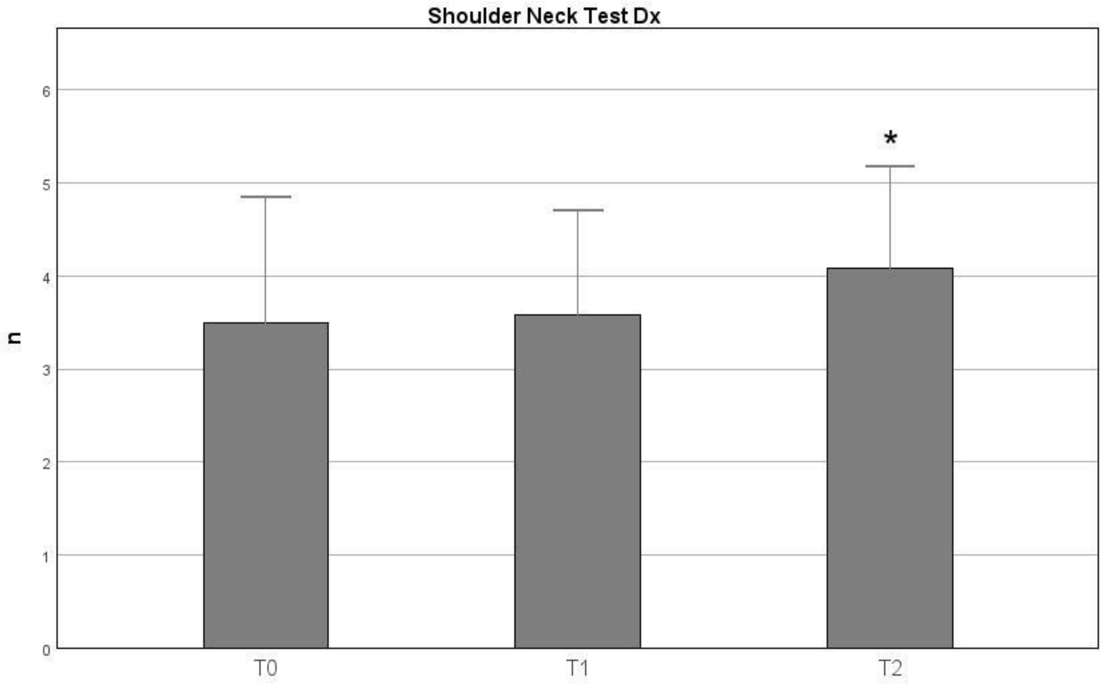

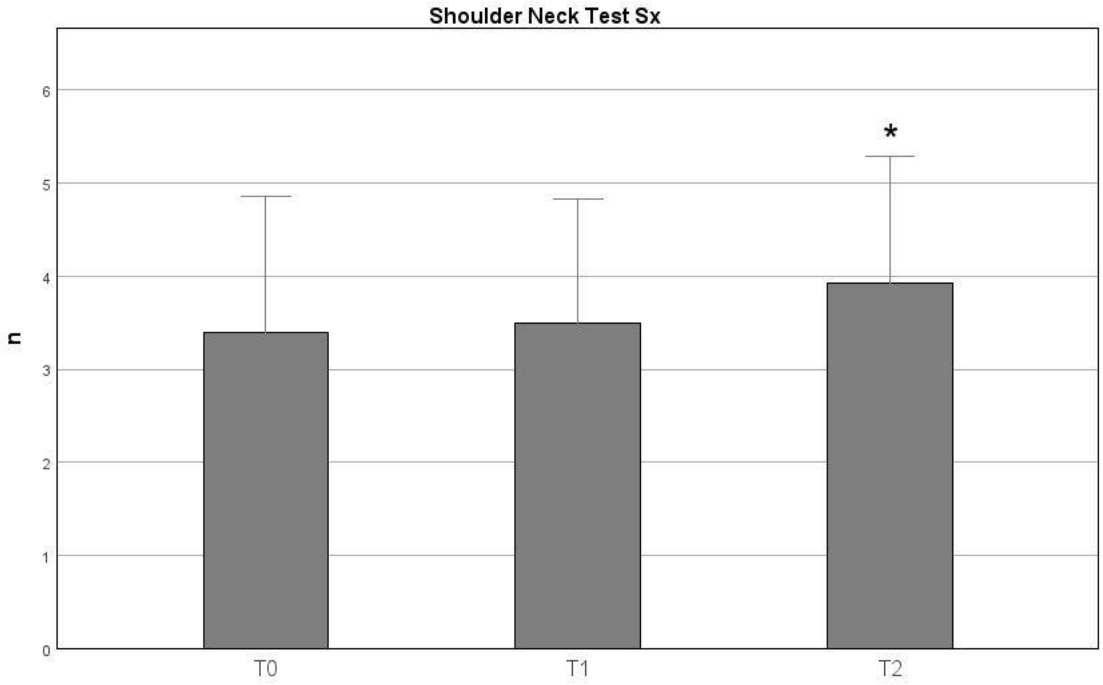

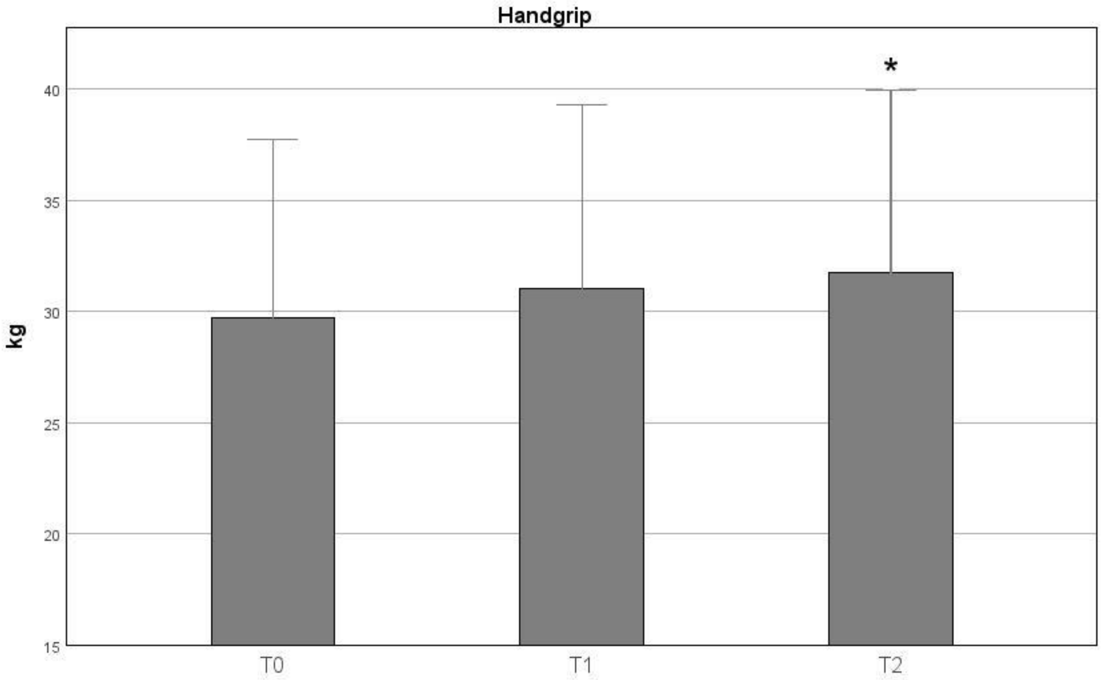
Handgrip
kg
40
35
30
25
20
15
*
T0
T1
T2

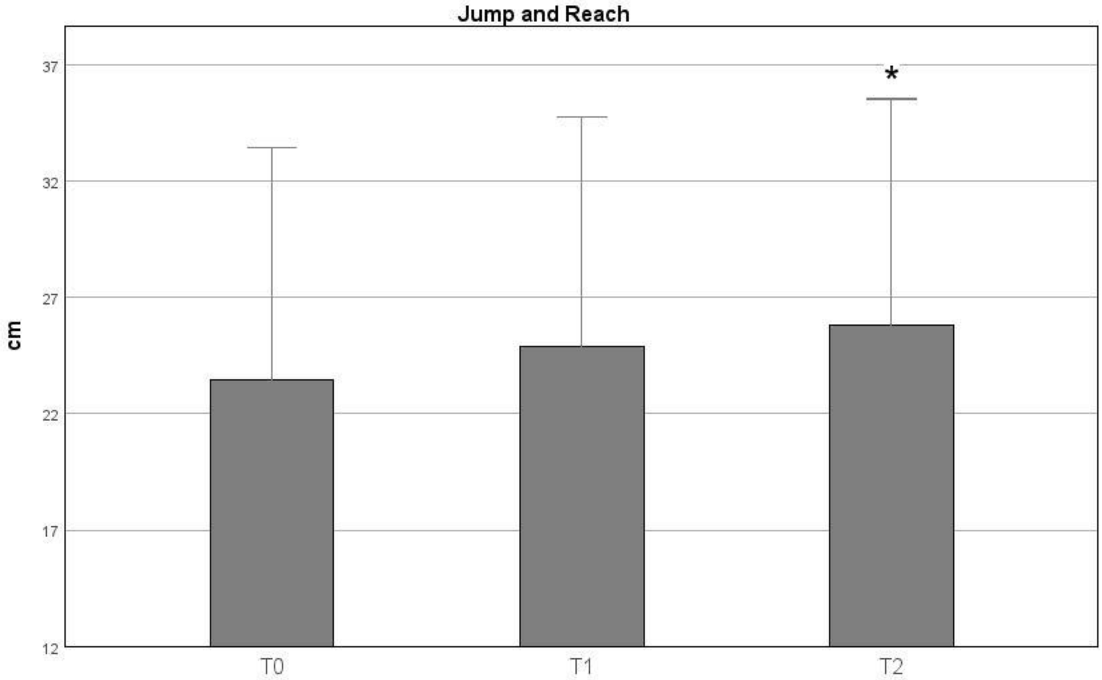
Jump and Reach
cm
37
32
27
22
17
12
*
T0
T1
T2

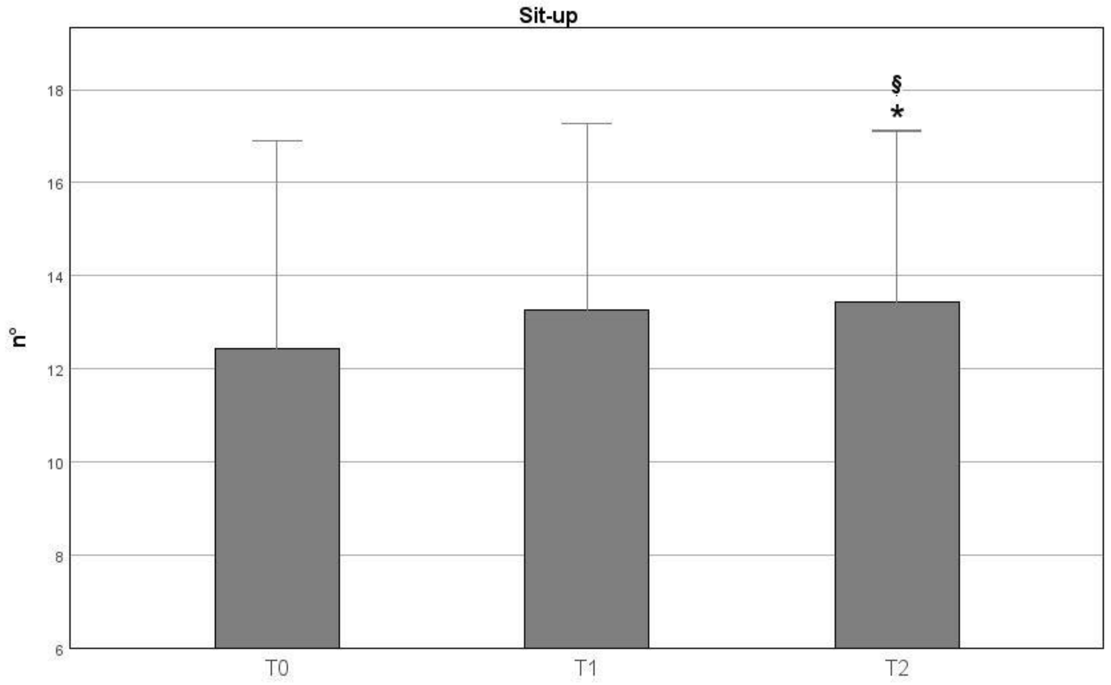
Sit-up
n°
18
16
14
12
10
8
6
§
*
T0
T1
T2

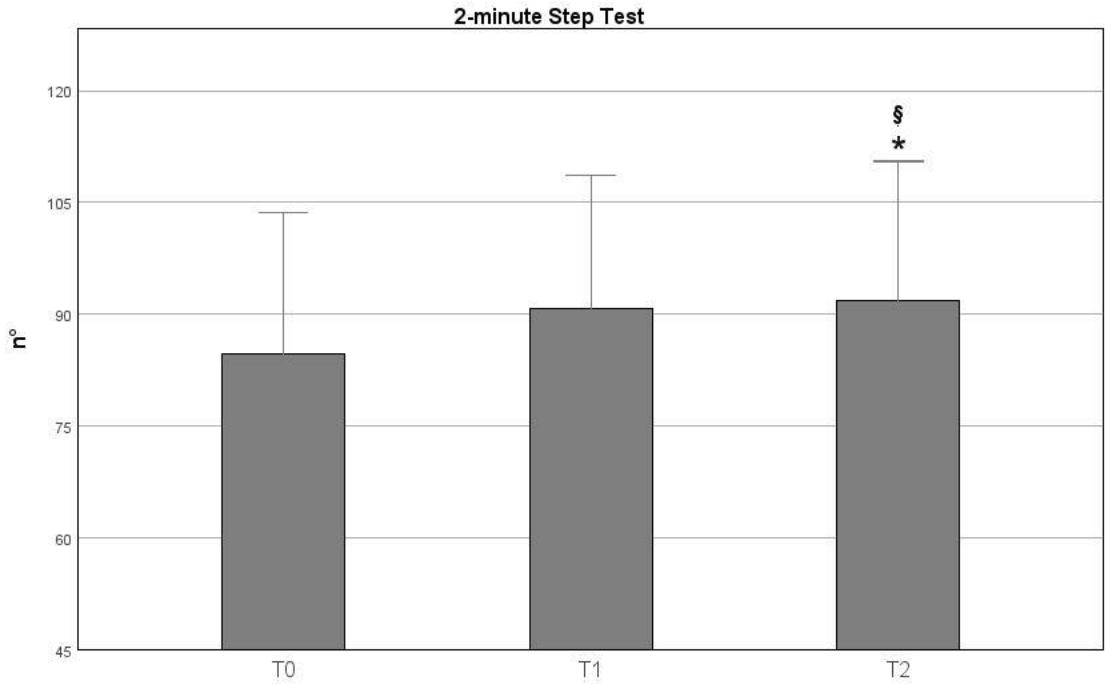
2-minute Step Test
n°
120
105
90
75
60
45
§
*
T0
T1
T2
